# “We Witches.” Knowledge Wars, Experience and Spirituality in the Women’s Movement During the 1970s

**DOI:** 10.1007/s00048-023-00359-w

**Published:** 2023-05-24

**Authors:** Anne Kwaschik

**Affiliations:** Professur für Wissensgeschichte, Fachbereich Geschichte & Soziologie, Universität Konstanz, Universitätsstraße 10, 78464 Konstanz, Germany

**Keywords:** Women’s movement, Witch discourses, Experience, Knowledge, Spirituality, Feminism, Frauenbewegung, Hexendiskurse, Erfahrung, Wissen, Spiritualität, Feminismus

## Abstract

During the 1970s, feminist activists reappropriated the figure of the witch in various ways as a symbol of alterity, political radicalism, feminist revolt or victimhood, or the presentation of subversive (healing or bodily) knowledge. The article investigates these witch constructions with a focus on its experiential foundations drawing on appropriations in Western Germany within a larger transatlantic history. First, it provides a brief overview of witch discourses in the 1970s, highlighting radical feminist, health-political and artistic milieus, based on representative Western European journals and movement literature. The article emphasizes the variety of witch images and its epistemic foci, showing that however different these approaches may appear, they all created women’s alterity. Second, the article examines alternative practices of knowledge production, focusing on health guides and advice literature, as well as on approaches to experience in consciousness-raising groups. This section demonstrates how witch discourses both enabled the movement’s knowledge empowerment, but were also part of complex boundary work within the milieus, such as in the debates about the relationship between experiential knowledge and theory. The last section shows how closely and in what ways spiritualist approaches were linked to this boundary work. The article argues that feminist milieus constituted themselves within the framework of feminist epistemologies against and within established knowledge cultures, thereby drawing further boundaries within the movement. In analyzing the “evidence of experience” (Scott) produced by witch discourses its overarching aim is to demonstrate that their historical relevance initially laid in its standpoint-creating character.

## Abstract (de)

In den 1970er Jahren eigneten sich feministische Aktivistinnen die Figur der Hexe in verschiedenen Kontexten und Konstruktionen an: als Symbol für Alterität, politischen Radikalismus oder politische Revolte, Repräsentation des verfolgten Opfers oder der alternativen Heilerin, die über subversives Körperwissen verfügt. Der Artikel untersucht diese Hexenkonstruktionen mit dem Fokus auf ihren Erfahrungsgrundlagen, wobei er sich auf Aneignungen in Westeuropa und insbesondere Westdeutschland in seinen transatlantischen Verflechtungen konzentriert. In einem ersten Abschnitt wird zunächst ein kurzer Überblick über exemplarische Hexendiskurse in den 1970er Jahren in radikalfeministischen, gesundheitspolitischen und künstlerischen Milieus gegeben auf der Grundlage einer Auswertung repräsentativer westeuropäischen Zeitschriften und Gesundheitsliteratur. Der Artikel hebt die Vielfalt der Hexenbilder und ihre erkenntnistheoretischen Zusammenhänge hervor und argumentiert, dass, so unterschiedlich diese Ansätze auch erscheinen mögen, sie alle Differenzmuster konstruierten. Daran anschließend werden im zweiten Abschnitt alternative Praktiken der Wissensproduktion analysiert mit einem Schwerpunkt auf Gesundheitsratgebern und consciousness raising groups. Es wird gezeigt, wie Hexendiskurse einerseits die Wissensermächtigung der Bewegung ermöglichten, andererseits aber Teil einer komplexen Grenzarbeit innerhalb der Milieus waren, etwa in den Debatten über das Verhältnis von Erfahrungswissen und Theorie. Der letzte Abschnitt des Artikels erweitert das Bild und verweist darauf, wie eng und auf welche Weise Spiritualität mit dieser Grenzarbeit verbunden war. Es wird diskutiert, wie diese Milieus sich im Rahmen feministischer Epistemologien gegen und innerhalb etablierter Wissenskulturen konstituierten und damit weitere Grenzen innerhalb der Bewegung gezogen wurden. Insgesamt demonstriert der Artikel mit dieser Analyse der „Evidenz der Erfahrung“ (Scott) in exemplarischen Hexendiskursen, dass deren historische Relevanz zunächst in ihrem standpunktbildenden Charakter lag.

When the rebellious and militant cry “the witches are back” rang out during the Walpurgis Night demonstration in Rome in 1977, it was by no means a positive return of the witch to the women’s movement, as some have read it (Graichen 1986: 24). In reality, this protest had assimilated discourses and references to historical images circulating internationally since the late 1960s, coalescing in the mid-1970s into more concrete political, self-reflexive and poetological as well as health and experiential-spiritualistic programs that were intended to mobilize non-hegemonic knowledge. These ideas would go on to reach a wider audience beyond the feminist movement, as in the staging of a large exhibition on witches in 1979 at the Hamburg Museum of Ethnology, which toured the Federal Republic of Germany until 1987, bringing nameless women back into history by placing their persecution at the center: A pyre on which books, slips of paper with names and numbers had been placed greeted visitors in the middle of the exhibition.^1^ Witchcraft also gained scholarly legitimacy and became a recognized object of research (Voltmer 2019).

The increased presence of the witch in the public debates of the 1970s and her inclusion in the wider linguistic and pictorial repertoire were understood by contemporary intellectuals as a return of the repressed in the context of the New Women’s Movement, or more concretely, using Roland Barthes’s terminology, as an “artificial myth”: “The best weapon against myth is to mythify it in its turn, and to produce an *artificial myth* (Barthes 1972 quoted in Bovenschen 1978: 90).” Indeed, the new witch allowed for the projection of a persecuted and rebellious female identity onto the past, through which contemporary feminists could determine their sociocultural place. Activists deployed the identificatory potential that lay in the various roles attributed to the witch, from victim of persecution to rebel, to diagnose structures of oppression and reveal the strength of women’s power in the present. Discourses of witchcraft in the early feminist movement—the focus of this article—must thus be understood as expressions of a consolidating and differentiating protest milieu. Furthermore, through the construction of these witch narratives, which referred back to the persecuted and repressed knowledge embodied by the witches, feminist protest, was both located in a long historical tradition and formulated as a claim to alternative knowledge.

Using the witch as an analytical category to explore feminist knowledge cultures allows us to consider their evolution and plurality as dynamic sites of struggles. Recent research in movement studies has shown how counter-knowledge and counter-expertise are produced within movements, exchanged and transmitted, how credibility is publicly contested and asserted, how new themes are established, and how new fields of knowledge and expertise emerge and occasionally reach the status of academic subject (Epstein 1998; Kirk 2007; Vettel 2006). This article argues that feminist milieus constituted themselves within the framework of feminist epistemologies against legitimate knowledge, thereby drawing further boundaries within the movement. These boundaries concerned conceptions of knowledge that, in critiquing androcentrism, questioned the very criterion of scientificity from the perspective of experience or spirituality as central expressions of knowledge empowerment via a recoding and reappropriation of the symbol of the witch.

The focus of the article is on the experiential foundations of witch constructions in feminist movements in the 1970s, in particular on appropriations and creations in Western Germany within a larger transatlantic history. First, it provides a brief overview of witch discourses in the 1970s, highlighting radical feminist, health-political and artistic milieus, based on representative Western European journals and movement literature that prominently used references to witches. The article emphasizes the variety of witch images and its epistemic foci, showing that however different these approaches to rebellion, liberation and victimhood or the reflections on alternative knowledge production may appear, they all created women’s alterity. Second, the article examines alternative practices of knowledge production and their experiential basis, focusing on health guides and advice literature, as well as on approaches to experience in consciousness-raising groups. This section demonstrates how witch discourses both enabled the movement’s empowerment, but were also part of complex boundary work among activists themselves within the movements, such as in the debates about the relationship between experiential knowledge and theory. Closely linked to this boundary work, as the last section of the article argues, were spiritualist approaches that intensified in Germanophone feminist milieus and discourses towards the end of the 1970s.^2^

The article thus ties in with research on feminist knowledge production (Haraway 1988; Scott 1991; Harding 2004; Tuana 2006; Kline 2010) and movement studies, which has, in recent decades, insisted on the centrality of knowledge to the understanding of new social movements (Eyerman & Jamison 1991; Della Porta & Pavan 2017). It revisits and expands the rich material on the reassessment of witch images within second-wave feminism that has thus far been researched primarily from a reception-historical perspective (Voltmer 2019; Wiedemann 2007; Wiedemann 2017). The overarching aim of this article is however to demonstrate that the historical relevance of witch discourses initially lay in its standpoint-creating character. As I have shown elsewhere, witch discourses served as a collectively shared matrix of knowledge empowerment, providing different (though closely linked) thought patterns while remaining receptive to new narratives (Kwaschik 2022). The present article develops these findings further by studying the experiential evidence of this knowledge empowerment, in line with Joan Scott, who, in her classic and controversial essay on the “evidence of experience,” underlined that claiming experience was (and is) not the representation of an existing identity, but rather the construction of a subject’s position, a standpoint, that is made evident by referring to experience (Scott 1991).

## Witch Discourses and Knowledge Wars

Critiques of existing knowledge hierarchies were crucial to the women’s movement, which presented itself as both a sociopolitical and epistemic project. In so doing, it intentionally challenged existing knowledge cultures in the name of female experiences that had been excluded from sociopolitical debates and academic institutions. Within the framework of a feminist epistemology, activists not only questioned the androcentrism of dominant knowledge cultures but also deliberately set alternative collective and experiential forms of knowledge generation against it (Haraway 1988; Harding 2004). Women activists promoted this knowledge revolution making it an integral part of their self-representation and political work. It led women to question traditional bodies of knowledge and the criteria of their production, as well as to demand new subjectivities, institutions and forms of science. In these contexts, the witch myth was prominently linked in and through feminist actualizations to the question of an alternative female knowledge production. The witch legitimized the quest for alternative and “buried knowledge,” representing herself as a persecuted knowledge culture that produced and disseminated knowledge about the female body and female health, in unity with nature. Her magic and naturalist knowledge was set in sharp contrast to the “system” of institutionalized modern science. In conceiving this alternative knowledge, activists co-constructed the category of “modern science,” despite the fact that it itself had emerged as an “implausible cosmology of mechanical philosophy” dependent on “occult forces,” as research on debates surrounding the scientific revolution in relation to witch hunting has shown (Easlea 1980: X). Against this backdrop, the witch became the “mythical term for the ancient knowledge and power of women,” as German journalist Gisela Graichen retrospectively states in her influential volume of conversations, *Die neuen Hexen* (The New Witches): “A witch is the wise woman, the herbalist, the healer, who has knowledge suppressed by historical persecutions and thus a strength and power within her to be uncovered (Graichen 1986: 25).”^3^

In this sense, the self-positioning in a historical succession of witches can be found in various political, spiritual, experiential, historical and medical contexts and often connects these aspects. Key to experiential evidence is the identification of modern women with the persecution of witches, a thought pattern alluded to by radical feminist writer and activist Shulamit Firestone, who in 1970 compared the religious and ecclesiastical persecution of witches with the political subordination of women. In *Dialectic of Sex*, she writes: “For example, witches must be seen as women in independent political revolt: Within two centuries, eight million women were burned at the stake by the Church—for religion was the politics of that period” (Firestone 1970: 15, footnote). In radical feminist circles, linking persecution (or even extermination) with political revolt became a prominent, characteristic rhetorical feature.

Similar arguments were used in the European feminist press of the early 1970s. In the German-speaking world, one of the first journals published by the autonomous feminist movement uses the witch as an example of anti-patriarchal self-empowerment. In its first issue, the *Hexenpresse* (Witches’ Press), a “critical organ of the second women’s movement” published between 1972 and 1976 in Basel, derived its political mission by drawing parallels between the “murder societies” of the past and the present: “DIE HEXENPRESSE appears at a time when the history of women begins. The name of the magazine recalls our prehistory, which is not yet over (Die Redaktion 1972: 4).” This appropriation of the witch continued to remain relevant even as the magazine’s position, at the end of the decade, moved toward mothers’ rights feminism.^4^

It is important to stress that the construction of this radical standpoint under the sign of the witch was directed not only against patriarchal structures, but also against milieus within the women’s movement that did not actively attack the chauvinism and antifeminism of leftist milieus in West Germany or Switzerland. These women uncompromisingly were held responsible for the current state of oppression on account of their willingness to be downgraded to being a “minor contradiction” and content with “being included” (“Einbezogenwerden”) instead of demanding emancipation (Feigenwinter 1973: 11, 13).

In sum, the evolution and meanings of the myth followed the evolution of second-wave feminisms by providing a matrix of a variety of narratives and images that were reevaluated and ritualized. Although this was clearly not a linear process, the focus at the beginning of the 1970s was primarily on radical political positions, similar to radical feminist activism in the United States. Although W.I.T.C.H. (Women’s International Terrorist Conspiracy from Hell), which emerged from the New York Radical Women group, only lasted a few years, its impact cannot be overestimated. Its activists did not content themselves with using the name, playing with its references and images, but experimented with the iconography of the witch in public protest performances, known as “zap” actions, which received much media attention—such as their first action “Up Against the Wall Street” on Halloween 1968 (Morgan 1970; Morgan 1977).

The mid-1970s saw a transition toward arguments that were more directly linked to the female body and toward reflections on the various connections between the different witch appropriations. The artistic-literary journal *Sorcières. Les femmes vivent* (1975–1982) (Witches. The Women Are Alive) illustrates this constellation: Its title refers to the witch as an (already established) symbol of past and present political rebellion that remains open to individual experience. However, in identifying historical and contemporary moments of persecution, the journal places female sexuality and female ways of existence at the center of the witchcraft discourse. Like the witches burned by the Catholic Church, the journal argues, thousands of women have been killed or mutilated by the “repressive apparatus,” dying “in general disapproval, stoned by society.” The reading deviates from a political interpretation and sets a new focus: “Because they had dared to live their body, to live their sexuality, to feel their body freely, because they had dared to enjoy (Gauthier 1975: 3).” The witch symbolizes female energy and creativity, underlining the journal’s aim of voicing feminisms that reassess visual creation and female artistic practices as part of its political agenda (Dumont 2014; Spencer 2019).

With the aim of rendering female experiences tangible, the Parisians participated in theorizing a new female language “to write with the body, with the totality of the bodily experience, and not only with the isolation of an intellect deluded about its mastery and inexperienced before life (Chawaf 1976: 5).” The artistic circles around *Sorcières* set the experience of difference linked to the female body and writing against hierarchized and institutionalized discourses. In promoting the “écriture féminine,” its authors considered “texts as smooth as a woman’s skin, where the meaning would be perforated and not set up as a system (Claude et al. 1978: 48).” This position qualifies as a kind of “no man’s land” “where everything remains to be said (Claude et al. 1978: 49).” Consequently, any scientific approach was rejected in this context. The knowledge of the *Sorcières* lies in the repressed, the unsaid—between the lines, in Luce Irigaray’s words, in the “inter-dit (Claude et al. 1977).”

Witchcraft discourses were related to different feminist milieus in the mid-1970s: They impacted on political and artistic narratives and contributed to the centering of the female body as self-evident part of the political agenda (beyond abortion rights) (Lenz 2008: 99, 107). For the feminist health movement in particular, they proved to be constitutive. The important booklet *Witches, Midwives, and Nurses: A History of Women Healers* (1973) signaled the continuities between the two states of persecution by printing the *The Malleus Maleficarum* in the annex. Embedded in the political arguments for women’s health centers and free clinics, the continuities between past and present persecution became part of a single political struggle. In other words: When Barbara Ehrenreich and Deidre English, the authors of the booklet, retraced the history of witches and of the popular health movement, they aimed to empower “what we might call the ‘women’s health movement’ (Ehrenreich and English 1973: 29)”.

In response to the oppressive character of the medical system, the goal of the women’s health movement was as radical as the political reflections in the *Hexenpresse*. They did not aim to include women in the medical profession, but demanded an opening up of “medicine to all women”:

“Men maintain their power in the health system through their monopoly of scientific knowledge. We are mystified by science, taught to believe that it is hopelessly beyond our grasp. In our frustration, we are sometimes tempted to reject science, rather than to challenge the men who hoard it. But medical science could be a liberating force, giving us real control over our own bodies and power in our lives as health workers. At this point in our history, every effort to take hold of and share medical knowledge is a critical part of the struggle—know-your-body courses and literature, self-help projects, counseling, women’s free clinics (Ehrenreich and English 1973: 42).”

Ehrenreich and English argued that the persecution of the wise woman was an intentional extermination organized by the learned medical profession to legitimize its political goals. First and foremost, the reference to the witches supported their main argument that “women have always been healers.” Medicine was part of women’s heritage: “our history, our birthright (Ehrenreich and English 1973: 3).”

Consequently, male domination was not the result of a longstanding, “natural” process but of “an active takeover by male professionals,” resulting in the political and economic monopolization of medicine and the control over institutions as well as medical theory and practice by men. In this narrative, the witch trials were evidence of the political partnership between the Church, the state and the medical profession, against the popular and experiential knowledge of the “wise women,” lay healers who worked for and with the people and the poor. Ultimately, the suppression of women’s bodies and their bodily knowledge were the result of the establishment of medicine as a male profession. Reclaiming this previously powerful position was a declared goal of the political struggle, which was also presented as a class struggle (Ehrenreich and English 1973: 4). However short the dominance of this historical image may have been, witch knowledge became the model of a self-determined approach to women’s bodies, women’s health and alternative medical practices, the effects of which continue to reverberate today.

The women’s health movement created far-reaching and distinct feminist discourses on women’s health and women’s bodies that were closely linked to its critique of patriarchy and domination, but shaped the national contexts in different ways. While in France self-help practices, and thus a distinguishable health movement, did not develop (Ruault 2023), in the United States, the new women’s movement established various independent outlets, founded self-help groups, specialized magazines, clinics and health centers (Nelson 2015; Morgen 2002; Kline 2010). In West Germany, the establishment of the movement was catalyzed by the mobilization against Paragraph 218 of the German Criminal Code, which prohibited and penalized abortion (Fourment 2023). The first Women’s Health Center in Western Germany (the FFGZ in Berlin) opened in November 1977 (Lauterbach et al. 1977). Self-examination and counseling groups began operation in 1973 and began publishing *Clio*, a self-help magazine, in 1976 in West Berlin.^5^

Beyond the narrower health feminists’ circles of the early 1970s, Ehrenreich and English’s book had an enormous impact on body-political and health-political readings and helped spark and disseminate ideas and narratives of a knowledge war.^6^ In this context, the romantic nineteenth-century myth of the wise woman proved to be adaptable for the 1970s women’s movement in several respects. The German philologist, jurist and mythologist Jacob Grimm had invented the Germanic wise woman as a representative of a pagan, non-Christian folk religion—a figure that remained influential through an emphasis on her medical knowledge. It was however the French historian Jules Michelet’s image of the witch “from the age of despair”^7^ that experienced a more direct, vivid and international reappropriation: Michelet’s midwife witch was a healer, rebel and victim all in one. As a helper of the common people, she distributed contraceptives and abortifacients, which were an important element for the women’s health movement. The fact that she lived and healed in close contact with nature, and also talked to trees, would become important for various strands of spiritual feminism (Voltmer 2019; Wiedemann 2007).

In reappropriating Michelet’s narrative of witches as independent healers and doctors of the common people, women activists in different milieus linked health politics with far-reaching knowledge-historical arguments in a simple, adaptable way. The idea of a knowledge war thus gained in importance beyond self-help circles and the emergent health feminist milieus. In Germany, the journalist Ingrid Strobl, who produced the bibliography for the German translation of Ehrenreich and English’s booklet, described the persecution as a “war against women” and pointed out that this war triggered a political consciousness-raising process. Directed against “the magic of midwifery and healing,” this monstrous persecution was characteristic of the patriarchal regime:

“It was a war against the female sex […]. The last regions still occupied by women, nature, and the female body, were to be taken, the last matriarchal remnants were to be finally destroyed. This war was fought with all ideological and material weapons. Ideologically, this war was a crusade against the ancient goddesses and their priestesses. Since time immemorial, women have been associated with magic, herbs, fertility spells. In matriarchal times, goddesses ruled the universe, they were the ones who gave birth to everything, who determined the course of nature, the cycles of life (Strobl 1976: 60).”

It is worth noting that this persecution was described as an act of extermination, meant to eradicate female knowledge and often presented as part of comparative victimology which included the experiences of other groups, such as Jews (Strobl 1976, 1977; Schwarzer 1988). The fact that the witch discourse in West German feminism occasionally reached the level of comparisons with the Shoah underlines the impact of genocidal readings of the knowledge war in these milieus (as well as the Shoah as the dominant commemorative structure).

Using a term coined in postcolonial contexts, this “war against women” was presented as an “epistemicide”—the murder and death of knowledge. “Unequal exchanges among cultures have always implied the death of the knowledge of the subordinated culture, hence the death of the social groups that possessed it,” as Bonaventura de Santos explains in *Epistemologies of the South*: “In the most extreme cases, such as that of European expansion, epistemicide was one of the conditions of genocide. The loss of epistemological confidence that currently afflicts modern science has facilitated the identification of the scope and gravity of the epistemicides perpetrated by hegemonic Eurocentric modernity (de Sousa Santos 2014: 149).”

Although feminist and postcolonial critiques of science share a critical view on the epistemic foundations of Eurocentric modernity and the construction of scientific objectivity as an instrument for maintaining power, health feminism discourses did not deploy the argument to per se dismiss science. While the rise and status of medical science, with its necessary rejection of other medical traditions, was questioned, it was clearly not science, magic or superstition that was at stake for activists, but power and economic privileges. Ehrenreich and English demanded that the “liberating force” of science be uncovered (Ehrenreich and English 1973: 42) inversing the historic roles of science and magic. Whereas the male doctor was like a “shaman, in touch with the forbidden, mystically complex world of Science […] Women health workers are alienated from the scientific substance of their work (Ehrenreich and English: 3)”; “in short, her magic was the science of her time (Ehrenreich and English: 14).”

In a similar way, self-help advice literature employed an alternative approach to bodily knowledge. In the book *Hexengeflüster. Frauen greifen zur Selbsthilfe* (Witches’ Whispering. Women Take Hold of Self-help; Frauenzentrum Berlin 1975), West Berlin self-help groups referenced witches in the title, its content and its illustrations: It quotes Firestone as a mission statement and provides readers with recipes from the “witches’ kitchen” alongside rich pictorial material. As the title implies, this experiential knowledge was whispered by the witches—in other words, passed on orally (among women) in secret (Schmidt 1988: 42)—and thus became the expression and product of the women’s “epistemic agency (Davis 2007: 124–141).” This whispered knowledge transmission thereby provides the potential for empowerment precisely because it was based on centuries of female exclusion from educational and professional sectors.

From a broader perspective, this alternative knowledge production and transmission was positioned as natural, experiential, authentic, and in sharp contrast to a male-dominated hegemonic knowledge and its techniques of examination and forms of expertise, as represented by conventional medicine and supported by a financial and commercial power complex. The topos of the medical and pharmaceutical industry—its money, power and influence projected back into the past—seems to be an emanation of the military and industrial complex that was at the core of leftist ideology critique of the 1970s, not least because it limited the freedom of science and dominated knowledge production (Schiller & Phillips 1970). Against this backdrop, the alternative witches’ knowledge clearly emerged as nature-bound, cautious, tentative and soft, epitomizing the counter-knowledge cultures of the 1970s (Stadler et al. 2020).

Be it in artistic Parisian circles, the American health feminist movement, or the Germanophone women’s movement, counter-knowledge appeared as an adaptable and contested resource for various social movements, and did indeed mean some kind of experiential knowledge: “The fact that the knowledges produced in these various instances are embedded in and embodied through lived, place-based experiences, means that they offer *different* kinds of answers than more abstract knowledge: knowledges that are situated and embodied, rather than supposedly neutral and distanced (Casas-Cortés et al. 2008: 42–43; emphasis in the original).” Rooted in a “shared cognitive system,” the invention of witch traditions can be understood as part of this experiential knowledge production, as “a way of thinking more adequate to their experience, a way of being which is more adequate to their daily struggles and needs, and developing appropriate organizational cultures (Cox 2014: 48–49).”

## Evidencing Experience: Questions of Alternative Knowledge Production and Science

The status of the “buried knowledge” of ancient times put the topic of alternative knowledge production on the agenda of the women’s movement, both in theory and in practice. Wise women had produced knowledge “without degrees, barred from books and lectures, learning from each other, and passing on experience from neighbor to neighbor and mother to daughter (Ehrenreich and English 1973: 3)” but the non-written transmission posed a serious reception problem in the 1970s. Herbs, to give a simple but basic example, seemed an entry point through which “to solve the problems directly related to our womanhood” independently of doctors and the pharmaceutical industry, “first for us personally, but also for the others.” Yet most books on herbs were likely written by men, as Heide Staaschen, co-organizer of the Hamburg exhibition recalled—although pharmacy lists from local archives sometimes helped to identify what was used in wise women’s recipes (Graichen 1986: 68). Since the “recovery of ancient knowledge” turned out to be a creative act, the challenge was threefold: the search for historical traces, the collection of present data and the production of new knowledge based on women’s experiences. Insisting on the “situatedness” of knowledges, alternative knowledge revolved around female experience, as a central epistemic principle aimed at transcending dominant power structures (Haraway 1988).

Thus, the solution lies in active knowledge work, collecting and transmitting female knowledge based on a reflective use of existing resources. The idea that knowledge production should begin with women’s own experiences involved different practices, such as the organization of open-space collective writing, the collection of experience-based data and material, collective self-examinations, and various forms of discussion groups. These practices were reflected in and performed through an internationally circulating body of literature consisting of companions and guidebooks, for which the exchange and analysis of experience were just as important as professional instructions or textbooks (Heinemann 2021). The health advice literature written by the early self-help groups that had emerged collectively and through their reflection on female bodily experiences, demonstrates the ways in which these “experiences” were engaged with. This literature, initially handwritten and handdrawn, sometimes stapled together, expresses the rebellious female reconquest of her own body, beginning with the choice of title. In 1970, the Boston Women’s Health Book Collective published a 193-page course book entitled *Women and Their Bodies*, which would become the mother of all advice literature in the 1971’s edition under the changed title *Our Bodies, Ourselves*.^8^ The West Berlin sister publication *Hexengeflüster* followed in 1975.^9^

The various brochures and guides presented themselves differently and had individual emphases, but they contained similar information on contraceptive methods, the structure and function of female reproductive organs, and natural remedies for venereal diseases. In instructing women on self-examination as well as abortion methods, these publications above all made a claim about the existence and significance of the female bodily experience. Clearly, these groups served as an alternative space of knowledge production, and the knowledge produced was as important to the women involved as traditional textbooks and authorized sources: “We learned what we learned equally from professional sources […] and from our own experiences. The facts were important, and we did careful research to get the information we had not had in the past. As we brought the facts to one another we learned a good deal, but in sharing our personal experiences relating to those facts we learned still more,” it says in *Our Bodies, Ourselves* (Boston Women’s Health Book Collective 1973: 2). Through uncompromising declarations opposing male medicine or, no less effectively, through the scandalous reports of experiences in hospitals or during gynecological examinations, recounted in the health feminist outlets such as *Clio*, women underpinned their claim to what Kathy Davis has called “epistemic agency” (2007), underlining the female capacity to critically reflect their experiences within the self-help literature. The authors of this advice literature reflected on the constructed nature of women’s experiences and the different discourses surrounding their bodies on an experiential basis (Davis 2007), without naively suggesting essentialist readings. Consequently, this literature should be read from a processual perspective, as the result of working with experience (and representing experience); reading it thus involves explaining a complex negotiation process including group members, readers and translators, and sometimes other feminist milieus

Working with experience took on different shapes, but did not necessarily entail a naive understanding of both the term and the process. Indeed, emphasizing the epistemic authority of experience meant problematizing the term and insisting on the need to unpack and historicize it (Murphy 2004). The women activists themselves led discussions interrogating the evidence of experience and its significance for the production of alternative knowledge. In the late 1960s, the status of experience was intensively debated and controversially theorized in the practice of consciousness-raising groups, as these groups first began experimenting with this epistemic principle and thus mapped future discussions.

The practice originated in radical feminist circles in the United States, in the New York Radical Women group founded by Shulamit Firestone and Pam Allen in 1967. It spread rapidly within feminist cells. Far from being a naive exchange of personal opinions, the meetings followed certain rules and agreed conventions (taking up practices from other radical movements) (Ruck et al. 2022). In this context, the evidence of experience was clearly a debated and contested result of group work, with occasional therapeutic elements, starting with individual experiences and the search for words to express something that had not previously been expressed, yet ultimately leading to a discussion of commonalities among the expressed feelings and experiences (Sobnosky 2013).

In the Federal Republic of Germany, women increasingly came together in “discussion groups” beginning in 1973–1974. Fighting against Paragraph 218 was undoubtedly an important catalyst, but soon the women activists worked on various topics: From the beginning, a working group at the Munich Women’s Congress addressed the issue of consciousness-raising groups (Wagner 1973: 145). By early 1977, there were reportedly around 70 groups in Berlin on topics such as neighborhood work, women at work, socialization research, Paragraph 218, self-help (self-examination) and self-awareness. The number increased not least because interested women usually had to actively start new groups since they could not join an already existing group (Lüders 1977). The German women’s magazine *Brigitte* published rules for discussion that were based on US experiences, which were certainly intended to broadcast the concept to a broader public in West Germany since the magazine addressed a readership primarily interested in topics such as fashion, psychology, relationships, and medicine. Psychologist Angelika Wagner (who participated in consciousness-raising groups in Ann Arbor, Michigan, where she earned her doctorate in 1971) explained, together with the participants of one of her groups in Reutlingen, the basic principles of the idea to a wider audience (Leeb 1974; Wagner 1973). Since the early 1970s, Wagner had played a major role in adapting consciousness-raising groups to the FRG through her publications and numerous teaching and group-founding activities. According to Wagner, working without a group leader, a group agrees on a topic at the beginning, listens to the women’s reports without interruption or comment, and then tries to “summarize the common aspects of the experience and relate them to our role as women in society (Leeb 1974; Wagner 1973).” Nonetheless, institutionalized groups also existed, such as in Frankfurt am Main, where the local Volkshochschule, an easily accessible institution of adult learning, offered six courses (*Frankfurter Rundschau* 4.9.1976).

Groups were founded on the assumption that women share experiences without knowing it, and that they should discover the structural character of their supposedly individual (and often demeaning) experiences in order to learn from each other. Through the use of empiricist rhetoric, this very experiential basis was presented as an alternative scientific tradition. As Kathie Sarachild, one of the leading US activists, stated,

“The decision to emphasize our own feelings and experiences as women and to test all generalizations and reading we did by our own experience was actually the scientific method of research. We were in effect repeating the 17th century challenge [of] science to scholasticism: ‘study nature, not books,’ and put all theories to the test of living practice and action (Sarachild 1978: 145).”

It is worth noting that this radical feminist critique of science did not reject the idea of science categorically, just as the feminist health milieu did not outright reject medicine (or science, as in Ehrenreich and English’s writings, when pointing to the magical science of witches). On the contrary, the movement’s knowledge was linked to alternative forms of self-proclaimed science in various ways. In the case of Germany, the sociologist Irene Dölling emphasized in retrospect the significance of consciousness-raising as an epistemic practice. In these groups, women generated shared and common knowledge that was based and validated (before and during the process itself) by personal experience, which lead to a different science:

“By means of self-awareness and the collective exchange of their own everyday experiences in their specific life contexts, women wanted to produce a common knowledge that transcended their individual horizons, based on which they could formulate interests and goals and act in an organized way. Scientific feminism was conceived as a building block in this production of knowledge, representing a different kind of knowledge than women’s everyday experiential knowledge. It was important for making visible links between individual practices and causes of contradictory experiences, living conditions, demands and constraints, but not separate from or superior to women’s everyday knowledge for the political movement (Dölling 2013: 32).”

Here, Dölling follows the political reading of experiential knowledge work within feminism. For her, as an Emeritus Professor of Women’s Studies, consciousness-raising (as described in Pamela Allen’s classic text about her group in San Francisco) was a multi-stage process that moved from personal experience to shared experience, to the analysis of that experience, and finally to political action (“opening up,” “sharing,” “analyzing,” “abstracting”) (Allen 1973).

The understanding of theory as emerging from women’s experiences and leading to social revolt was characteristic of radical feminism; and was shared by the theorists of the Mouvement de libération des femmes (MLF) in France. They recognize that science and theory were sites of women’s oppression. Nevertheless, they launched the journal *Questions féministes* (Feminist Questions), as journal of feminist theory, in 1977, on the basis of an expanded and feminist conception of theory that left social elitism behind: “Any discourse, whatever its language, that attempts to explain the causes and operation, the why and how of women’s oppression is theoretical […]; it is any discourse that attempts to draw political conclusions (*Questions féministes* 1977: 3).”

In a programmatic way, *Questions féministes* claimed a feminist science that paralleled female existence and was determined to restructure power relations in society (especially in fields such as medicine, gynecology, psychiatry, psychology):

“We wish for a feminist science to emerge that can account for the formation of patriarchal hierarchies (and its impact on individuals), and thereby modify the global analysis of society. The interest of this feminist science is very quotidian: the emergence of subversive feminist discourses has allowed us and continues to allow us to modify the course of our existence. But in addition, the question arises as to know how a feminist point of view can intervene in those fields where a series of powers aiming at the reproduction of patriarchal structures is directly exercised (*Questions féministes* 1977: 4).”

However, there were contradictions within the claims made by different groups. Whereas for Allen, experiential knowledge becomes an intellectual experience through the application of theory and shared analysis, other women’s groups preferred to avoid abstractions. As discussed in the previous section, the *Sorcières* in Paris insisted on their alternative and open-space feminism. From their perspective, theory in any form was suspicious, part of legitimized hierarchal discourses that had alienated and would further alienate women from their experiences. In West Germany, the psychologist Wagner represented a similar position, though it was anchored in a different framework. She opposed the abstraction of personal experience and influenced many self-help groups through her rather psychological understanding of “experience,” as recent research emphasizes (Ruck et al. 2022; Lux 2019). Following this line, the Freiburg women’s group, for example, which split off from a left-wing group at the Pädagogische Hochschule (College of Education) in October 1972, focused on role constraints, on overcoming fear in and through group experience: “Don’t say ‘one,’ say ‘I.’ Generalizations make the conversation impersonal (Lux 2019: 60).” Evidently, the goal of the groupwork was also to gain awareness of one’s own personal experiences, but not as an “experience of abstraction,” in which the experiences of group participants enter in an abstracted form and are mediated through existing knowledge about society and its institutions, as described in the concept of “epistemic agency.” On the contrary, these women activists understood “theory” as an obstacle to feminist consciousness-raising. This position, too, was not without opposition. It fell victim to critiques and parodies within the women’s movement, including in the articles of *Schwarze Botin* (Black Messenger). The fixation on personal experience and thus on an “apparent given identity” was seen as a “behavioral cliché of the new women’s movement,” parodied and satirically exaggerated (Goettle 1978, quoted in Lux 2019: 65). The two positions mark different practices of self-awareness: While, on the one hand, structures of women’s oppression are to be made available for the political movement through the inclusion of one’s own experience in a collective process, the other group seemed more concerned with the direct changes in consciousness through emancipation in discussion groups.

The sharp distinction between the two approaches seems slightly excessive however. On the one hand, Wagner’s approach differed from most therapeutic approaches due to the deliberate exclusion of external expertise. She explicitly distanced her approach from therapy insofar as the experiences presented were not interpreted against the backdrop of an individual life story but within its sociopolitical context. In addition, the group discussion aimed to reassemble the different parts of the experience “like in a kaleidoscope” to further foster solidarity among women—which could be understood as a political goal (Wagner 1973: 146). On the other hand, the question of how far the abstraction of one’s own experience reaches was, in practice, an object of permanent negotiation *within *the groups. Since it was not always possible to separate those who prefer reading Marx and Engels from those who need to “talk about themselves, experience themselves” (Stefan-Blees 1977)^10^ into individual groups, this question divided the groups from within, but also forced their development through the negotiation of the experiential approach, as numerous memoirs and group meeting minutes show.^11^

In light of these different approaches, it can be summarized that the meaning of experience for consciousness-raising processes was at the core of group discussions and the movement’s internal debates. However, from a broader perspective, what was at stake here was the relationship between experience and theory. While dialectically, the goal of the awareness-raising process is the generation of theory, a more psychological approach opposes all theory building, and categorically separates it from “experience (Wagner 1973: 156).”

## “The Witch in Us”: Witch Knowledge and Spirituality

Witch myths include the aura of the demonic and mysterious, in direct contact with nature, as well as the notion of magic powers (Bennewitz 1996). During the 1970s, historical connections were made with ancient practical knowledge about herbs and natural healing methods, yet witchcraft knowledge also evolved to become synonymous with spirituality, pointing to practices of living and knowing that transcended the traditional technologies of the self. In the few but influential in-depth studies on the Federal Republic of Germany, spiritual phenomena are most often located within the context of religious-historical framings and periodized within the spiritual boom during the New Age (Eitler 2007), without emphasizing feminist legacies (although contemporaries such as Fritjof Capra insisted (not without cliché) on the significance of feminism to spirituality) (Jung & Kaffer 2014). However, a look at witch knowledge shows that the women’s movement brought its own traditions, narratives and strands of interpretation to this phase, the specifics of which the literature has yet to account for in a systematic and comprehensive way.

Indeed, the preoccupation with spirituality in the late 1970s should depart from debates within the movement despite the skepticism among many feminist cells. As the feminist press reported, these phenomena, which gained in importance notably in the US and the UK, were dismissed as apolitical and reactionary substitutes for religion, or criticized because of the prominence of maternalism (Rheinz 1979). Nonetheless, this in and of itself did not indicate a clear boundary between feminism and spirituality. Although accusing spiritual feminisms of encouraging apolitical positions, the women involved did not question that the rejection of patriarchal categories of space and time or prohibitions of thought and feeling could include or lead to accessing other levels of reality. In the words of *Courage:* “‘Spirituality’—for lack of a better word—includes, among other things, exploring the meta-physical, spiritual dimensions of reality, its forces and ‘laws,’ the potential of spirit-activity, whereby spirit-activity means the total before emotion/intuition/intellect/will (Kahn-Ackermann 1979: 26).”

The caution evinced in terms of using the word and the search for other terminology is characteristic of the contemporary discussion surrounding spiritualism. The term “spirituality” was rarely used without delimitations, restrictions, or new interpretations. Moreover, its use was uncertain, and it indicated that the phenomenon triggered a negotiation process within and between groups. Sometimes referring to yoga and meditation practices (Elsner 1984: 403), it was often controversially discussed in the groups and rejected because of its supposedly non-political thrust. It nonetheless played a role early on in connection with the witch symbol. Furthermore, the symbol of the witch shows that the juxtaposition between political and spiritual feminisms obscures the multi-layered nature of contemporary discussions. In this sense, it symbolized the recovery of forgotten self-knowledge, namely the knowledge of the wise women, and *differs* from spiritual knowledge as a product of male knowledge/power structures. In other words, the expansion of women’s spaces of experience and agency transcended the divisions between the political and spiritual spheres. At the 7th Women’s Summer University in Berlin in 1983, Cornelia Elsner argued:

“For me, the struggle for self-determination as a woman means not only taking on important social-public spaces and positions, but also (re)-discovering possibilities that lie dormant within me. That is, to acquire knowledge and knowledge from which we have been split off and alienated. This means both modern scientific and humanistic knowledge as well as earlier spiritual knowledge of women—such as wise women, so-called witches. If we consider the social position of these women and the struggle of the patriarchy against them, it becomes clear that the development of spiritual qualities and their use are by no means without socio-political effect (Elsner 1984: 403).”

In a similar way, Doris Stauffer, co-founder of the Women’s Liberation Movement (FBB) in Switzerland, saw “spirituality arising” from the memory of the “buried knowledge” and repressed individual powers:

“I myself use >spirituality< carefully. I prefer to paraphrase what I mean by it with >if you listen to yourself< or with >hexenwissen< [witches’ knowledge] or >intuition<. Because what women understand by spirituality today has nothing in common with that prudish, sanctimonious spirituality which, because it is spiritual, must exclude the body according to male, dualistic logic. It also has nothing to do with Judeo-Christian ideas of religion […] or other >there is only one god and he is male< religions. Spirituality is perhaps what we are slowly developing within ourselves as we begin to remember our very own forgotten knowledge (Stauffer 1983).”

In this context, the term “witches’ knowledge” seems apparently more plausible and useful than the controversial though established term “spirituality,” which does not adequately relate to female experiences. In Stauffer’s reading and as reflected in her teaching and artistic methods, “witches’ knowledge” belongs to processual practices of self-emancipation, producing self-confidence and releasing artistic creativity. From 1977 to 1980, Stauffer, who co-founded the independent F + F Schule für experimentelle Gestaltung (F + F School for Experimental Design) in Zurich in 1971, organized and conducted artistic “Witches Courses” that served as an experimental space for self-awareness, personal development through artistic practice, and group discussion.

The first course description emphasized the implementation of “our ideas of creativity, our own language, our concerns and what we have to share as women” and indicated “information, discussion, practical work with different materials (spatial, areal, video, photo, etc.)” as ways of working (Koller and Züst 2015: 122). In Stauffer’s life story, these artistic experiments evolved into practices that were ultimately connected to cults and spiritual awakening. From the early 1980s onwards, when she experienced identifying with the independence of the witch as an act of self-liberation (Stauffer 1983), she engaged in pagan and matriarchal rites and celebrated female seasonal festivals such as Walpurgis Night or Summer Solstice, beginning in 1983 or 1984 in larger circles and then continuing until 1991 in smaller groups (Koller and Züst 2015: 175).

It is worth noting that the spiritual dimensions of feminism have several origins and were found in groups and discussions on health feminism and technologies of the self or maternal theology. As Stauffer’s example demonstrates, feminist subcultures that were detached from traditional political protest created alternative spaces which were open to and included new forms of authenticity. Research on the subjectivity regimes of the 1970s has extensively discussed how technologies of the self developed during that decade, paving the way to the New Age experiences of the 1980s (Eitler 2010). Indeed, the journey to the self triggered different means of inner transformation, with its associated holistic imaginaries and ideas about new communicative spaces, self-actualization rites and group creativity, to name just a few aspects (Reichardt 2014).

In the case of witches, the search for authentic experiences led activists to uncover deeper layers of consciousness and spiritual forms of self-awareness, including the return of repressed forces and energies. Experienced as a recollection or a reconnection with older traditions and forms of knowledge, it signified the moment of “recognizing through the witches what is being done to us (Graichen 1986: 25).” On so-called “Witches’ Weekends,” the reference to one’s own everyday life was established, and the feeling of being oppressed, of being “devalued and restricted as a woman,” was discovered as a common element of female experience (Graichen 1986: 24). But the process of identification, as in Stauffer’s artistic milieu, was not only determined by persecution but also by empowerment, as spiritual groups emphasize: “The power rests within us. We must awaken it and begin to live it (Graichen 1986: 58).”

In addition to the subjectivity regimes, the body-based knowledge production within the women’s health movement was crucial for the evolution and practice of spirituality. Health feminism encouraged women to question their body feelings and to search for new ways of wellbeing, and the very practical organization of physical self-examination and other body care offers opened up experiential spaces for new and authentic bodily experiences. The example of the Berlin self-help store “13th Moon” demonstrates the dynamics of how a politically motivated self-help initiative became a health and wellness center, with offerings that supported or favored more spiritual experiences. Founded in 1976 by a group of women who had been active in the self-help movement since 1974, the store’s goal was to reach a wider public than was possible with a self-help group.^12^ At the store, in addition to several self-examination groups and a birthing group, there was also an herbal group, which was given the task of “reappropriating” the knowledge of the wise women. The herbal group created an herbal card index, experimented with teas and skin care products, and collected testimonials from women who had used herbal remedies.^13^ However, as the topic of “health” quickly diversified, more groups were created—in the short-lived project’s heyday there were four massage groups, a bioenergetics group and two nutrition groups that dealt with prevention and healthy eating “on a scientific (biochemistry) and political level.” Relaxation and breathing exercises were on the agenda, with the aim of gaining more knowledge about one’s body and thus greater self-esteem.^14^ Whereas, in this context, spirituality was not a declared goal, these groups provided new experiences with the female self that were different from the conversation groups.

The example of the “13th Moon” is enlightening for understanding the debates surrounding the supposed depoliticization of women’s health issues at the end of the 1970s. In the store’s case, new groups joined in, marginalizing the founders, and finally excluding them in June 1977: Self-help as a political issue began to lose in importance, and most women turned to other health issues, such as ecology, nutrition, relaxation, massage—only the home birth group remained active. Eventually, the conflict made the founding women withdraw: “It no longer comes across that we are part of a women’s movement, which would mean that we keep centering our specific oppression as women as the starting point of our efforts.”^15^ Body awareness and alternative forms of healing lost their political meaning, disappearing into alternative treatments and clearly spiritual exercises.

The example of the Schiran women in the late 1970s illustrates how health feminism was translated into an independent spiritual and shamanic lifestyle that nonetheless maintained its political aspirations. Ute Schiran initially studied medicine, becoming involved in the women’s health movement and, in 1979, co-founding the Munich “Beratungsstelle für natürliche Geburt und Elternsein in München e. V.” (Information Center for Natural Birth and Parenthood). From 1980 onwards, she lived in a self-sufficient community with other women, who called themselves the Schiran women, first in Lower Bavaria, then in the Eifel, in Cornwall, and other places, and finally in the Swabian Alb. The development of the group’s spirituality can be described as a path of initiation. First, five-day courses were offered to help women find their way back to their own roots with the help of trances, before the group itself began to work with rituals (such as the Demeter Festival) and finally opened a school of magic. Teas, massages and herbs were still part of the process, as were self-healing aspects related to previous self-help activities, but at the core of the group work were now ritual acts on the path to self-knowledge and the realization of one’s own duty (Graichen 1986: 255–274).

It should be emphasized that the Schiran women rejected the term “spirituality” to identify their culture and lifestyle, not least to distinguish themselves from New Age cultures. They did not favor the term “witch” either, although some Schiran women had founded a witch group within the women’s movement. However, identifying with the knowledge of the wise women remained important, even if it was replaced by the impact of matriarchal cultures as the central historical reference. Thus, instead of “spirituality,” there was talk of magic as a specific female approach. This establishment of distinct female spaces of experience, which were ultimately also dedicated to earth healing, can be understood as a contribution to the women’s movement, to which the Schiran women still felt they belonged (Graichen 1986: 255–274). The tremendous “force field of common energy for our un-folding” or in other words “the ecstasy [sic] within women’s contexts” was clearly and intentionally directed against a male standard (Schiran 1983: 486).

The pursuit of this lifestyle by the Schiran women was certainly a prominent and far-reaching example of spirituality in close connection with the women’s health movement. The impact of matriarchal theology, however, proved significant more broadly. In particular, the works of the feminist theologian Mary Daly influenced Western European feminism at the end of the 1970s, and introduced or enhanced spiritual thought patterns, including those of the aforementioned Swiss artist Stauffer or the radical feminist editor of the *Hexenpresse*, Gunild Feigenwinter, whose texts on mothers’ rights took on a spiritualist language in the early 1980s, summoning the invisibilization of cosmic-feminine forces by the “phallic logos (Feigenwinter 1983).”

In her seminal book *Gyn/Ecology* (1978), Daly linked the excavation of matriarchal traditions with the realization of a radical feminist “be-ing.” She prominently used scientific rhetoric, to describe her project of a counter-science, namely the “science, that is the process of know-ing, of ‘loose’ women who choose to be subjects and not mere objects of inquiry (Daly 1978: 10).”^16^ The book took up the dialectic of the witch myth, alternating between victim and rebel, and marking this difference with different terms, using “witch” for the object and victim, and “hag” for the rebel and subject of history:

“For women who are on the journey of radical be-ing, the lives of the witches, of the Great Hag of our hidden history are deeply intertwined with our own process. As we write/live our own story, we are uncovering their history, creating Hag-ography and Hag-ology. […] Women travelling into feminist time/space are creating Hag-ocracy, the place we govern. […] The vehicles of our voyage may be any creative enterprises that further women’s process. The point is that they should be governed by the Witch within—the Hag within (Daly 1978: 15).”

From the beginning, Daly used the witch as a symbol for a radically alternative and subversive knowledge that developed over the years into an ontological category (Wiedemann 2007: 283–287). However influential this radical critique of Christian theology and Cartesian methodology was, Daly was not linked to existing cults such as the contemporary Z. Budapest or Dianic traditions, which at that time already dominated and influenced the image of spiritualism.

It may be noted that postwar feminism and spiritualism were intricately linked in their definitions and practices, which became more differentiated and specific during the 1980s. The figure of the witch in this context, however, did not lose its impact, but was still used to emphasize the specifically feminine, as during the Women’s Summer University in Berlin in 1983, when Cornelia Elsner insisted on the need to find a synthesis between spiritualism and feminism—by linking the “changes within us *and* the power relations of the patriarchy (Elsner 1984: 404, emphasis in original).”

To conclude, this article has highlighted the experiential basis of feminist knowledge production and used the witch myth to access the pluralistic knowledge cultures within this movement. It has shown how witch knowledge served as common experiential basis in different contexts, which attributed revolutionary power, the command of practical medical knowledge, and body-bound artistic practices of alterity to the witch. Be it in artistic writing collectives, vaginal self-examination groups, or spiritualist meetings, practicing the evidence of experience was a central feminist “discursive and social technology (Murphy 2004: 124).”

When the Walpurgis Night demonstrations spread internationally after 1977, the witch claims became shared protest practices; through the costume, the candle-carrying, and the common shouts of struggle, a whole arsenal of emotions and images was mobilized, the content and historical relevance of which paled in comparison to the power of international protest. Thus, witch knowledge helped to “foster the coordination of disconnected, local, and highly personal experiences and rationalities within a shared cognitive system able to provide movements and their supporters with a common orientation for making claims and acting collectively to produce social, political, and cultural changes” (Della Porta & Pavan 2017: 300).

It is not surprising, however, that throughout the 1970s questions of alternative knowledge production became crucial to social movements. In the post-industrial societies of Western Europe, which had already adopted the habit of describing themselves as knowledge societies, “knowledge” was approached as a central resource, not only in public debates and the presentist sociological literature, but also in emancipation struggles for social recognition and political legitimation. In this context, knowledge production in the movements proved to be a collective process of negotiation between different existing knowledge repertoires, on the one hand, and experiences of the activists in the movement, on the other.

The process of experience-based knowledge production has been approached as a practice in which women in different milieus negotiated between multiple different existing discourses and their experiences. While women activists shared experiences that conformed to existing discourses due to their socialization, they were also faced with discordant and contradictory experiences that led to disagreements and division. Thus, this knowledge empowerment must be considered a “conflictual dialogue (Cox 2014),” in which demarcations were drawn or contradictions reconciled. Including “abstraction” on a theoretical level, for instance, was obviously experienced by some women as a form of pressure or alienation, whereas for others a theoretical approach was a necessary instrument for understanding personal experiences. In a similar way, the relationship between feminism and spiritualism became an object of controversy.

However, it should be noted that it was these gaps and demarcations between several discourses on the one hand and between discourses and experiences on the other hand, that allowed women activists to develop a critical and reflexive discourse and to exercise their “epistemic agency” in alternative spaces such as the consciousness-raising and self-help groups, the writing collectives, resistance camps, or women’s universities. From a broader perspective, these developments led to what the literature refers to as a “knowledge revolution,” not only in terms of the production of new bodily knowledge by women for women, with the aim of changing social norms on reproduction and sexuality, but also, more fundamentally, as an “epistemic revolution from the ‘other sex’ to women as individuals to becoming the individual subject of one’s own sexuality and relationships, in the sense of redeeming modernity’s promise of self-determination, liberation and equality (Lenz 2008: 99, 100).” The confrontation with the witch marks a crucial moment in this process on two levels—that of inner transformation and that of cultural change.

## Acknowledgements

I am greatly indebted to the inspiring discussions with the “Movement Knowledge” working group at the University of Konstanz in 2021. I would like to thank the two anonymous referees for their close readings and extraordinarily careful reviews. The article originates from a joint research project with Emeline Fourment (University of Rouen) on health feminism (see our special issue Critique international 2023, No. 99). My thanks also go to the staff of the Women’s Research, Education and Information Center (Frauenforschungs‑, Frauenbildungs-, und Fraueninformationszentrum, FFBIZ, Berlin). Dagmar Nöldge’s knowledge and kindness in advising researchers was extremely helpful in finding witch material.

## Footnotes

1 See the interview with co-organizer Heidi Staschen, www.deutschlandradio.de/archiv/dlr/sendungen/merkmal/258094/index.html. Staschen notes that reading Ehrenreich and English was instrumental to her interest in witches (Eichhorn 2006: 259).

2 Within this focus, however, the article cannot pretend to be more than an initial foray into a transnational knowledge history of the witch symbol. After the period under investigation here, witch discourses developed further, as spiritual milieus diversified over the course of the 1980s and re-readings of Marxist theory on reproduction and capital accumulation became influential.

3 All translations from the German or French are the author’s own unless noted otherwise.

4 The complete run of the journal is accessible at the FFBIZ.

5 To date, systematic research on the history of the women’s health movement in the FRG has only begun, Fourment (2023). For a collection of materials by the Feminist Health Research Group in West Berlin, see Bonn und Zimprich (2018); short notes about the centers in Stolzenberg (2000); on advice literature, see Heinemann (2021).

6 This observation culminates in Heinsohn and Steiger (1985), who claim that every witch hunt was directed against a body of knowledge and not against a gender.

7 The widely used term “age of despair” comes from the introduction of Michelet’s famous work (1862), which suggests that this age coincided with the image of the witch. In the 1970s, the journal *Sorcières* refers explicitly to Michelet in its first issue, but the reception by Marguerite Duras was equally important, see Gauthier (2002); *Sorcières* (1, 1) (1975) (back cover). For quotations see, for example, Gauthier (1975); Siebenschön (1977).

8 Roesch (2021: 183–191).

9 Research on the side effects of the pill had already culminated in *Frauenhandbuch Nr. 1. Abtreibung und Verhütungsmittel* (Women’s Handbook No. 1. Abortion and Contraceptives; Brot und Rosen 1972, 1974).

10 *Tagesspiegel*, 06.02.1977. I used the newspaper clipping collection in FFBIZ A Rep 20 (7) Selbsthilfegruppen.

11 See the minutes of the women’s group (Konstanz) 07.06.1977, written by Kerstin. FFBIZ A Rep 20 (7) Selbsthilfegruppen.

12 Ladenclinch im SH-Laden „Im 13. Mond,“ 7/77. FFBIZ A Rep 400 Berlin 2014. 7–14d; 20.15. (1)

13 „Zur gegenwärtigen Situation im 13. Mond“ *Eingriffe Extra*, April-May 1978, No. 10/11. FFBIZ A Rep 400 Berlin 2014. 7–14d; 20.15. (1)

14 Ibid.

15 Ibid.

16 Daly uses active verbs in order to stress that “the journey of women becoming” is a “spiraling creative process,” “that can break the brokenness of the ‘fields,’ deriding their borders and boundaries, changing the nouns of knowledge into verbs of know-ing (Daly 1978: 11).”
